# Canine babesiosis: from molecular taxonomy to control

**DOI:** 10.1186/1756-3305-2-S1-S4

**Published:** 2009-03-26

**Authors:** Peter J Irwin

**Affiliations:** 1 Australasian Centre for Companion Animal Research, School of Veterinary and Biomedical Sciences, Murdoch University, Murdoch 6150, WA, Australia

## Abstract

Canine babesiosis is a clinically significant emerging vector-borne disease caused by protozoan haemoparasites. This review article considers recent literature pertaining to the taxonomic classification of *Babesia *and *Theileria *species affecting dogs and the geographical distribution of these parasites. The diagnosis of canine babesiosis by traditional, molecular and serological methods is reviewed, together with recent advances in our understanding of the pathophysiology of piroplasmosis, and of the treatment and prevention of this disease.

## Introduction

Canine babesiosis is a common and clinically significant tick-borne haemoprotozoan disease with a worldwide distribution. *Babesia *species are often referred to as piroplasms, a collective term for phenotypically similar protozoan parasites that utilise mammalian erythrocytes in their life cycle. Piroplasms of domestic animals encompass two main genera, *Babesia *and *Theileria*, and have been subject of intense research interest and molecular-based re-classification during the last 10 years. In dogs, infection by these haemoparasites results in a wide range of clinical presentations; from subclinical disease to serious illness characterised by fever, pallor, jaundice, splenomegaly, weakness and collapse associated with intra- and extravascular haemolysis, hypoxic injury, systemic inflammation, thrombocytopenia and pigmenturia [1-3]. Although canine babesiosis is recognised as a tick-borne disease, transmitted by a variety of well-described Ixodid vectors around the world, *Babesia gibsoni *is an emerging disease with molecular evidence of clonal expansion due to non-vectored transmission by blood exchange during fighting and biting [4-6]. Recent research into canine babesiosis has focussed on determining the taxonomic status of well recognised and newly discovered canine piroplasms, developing improved diagnostic methods, investigating aspects of pathophysiology and searching for improved chemotherapeutic and immunoprophylactic protocols.

## Taxonomy and molecular phylogeny

The classification of *Babesia *spp. places them in order Piroplasmida within the phylum Apicomplexa. Two morphologically distinct forms of the erythrocytic stage in the canine host were recognised in early studies that led to the naming of the larger form, measuring approximately 3–5 μm as *B. canis*, and the smaller (1–3 μm) as *B. gibsoni*. Despite painstaking observation of the parasites in blood films by many early researchers, further understanding of the taxonomic classification of these parasites was hampered for the best part of one hundred years by the fact that within these general size ranges, their morphological features did not permit further differentiation. The first suggestion that all *B. canis *isolates were not identical species came from the German protozoologist Eduard Reichenow who recognised differences in pathogenicity of "*B. canis*" isolates from France and North Africa (these were most likely parasites that are currently known as *B. (canis) canis *and *B. (canis) vogeli*, respectively) [7,8]. Further clarification did not come until the late 1980s with the advent of molecular tools for phylogenetic studies; molecular genotyping of canine piroplasms has resulted currently in the identification of four large and at least four small parasites, but it is likely that new species will be added as further isolates are characterised.

"*Babesia canis*" was reclassified into three sub-species (*B. canis canis*, *B. canis rossi *and *B. canis vogeli*) on the basis of cross-immunity, serological testing, vector specificity and molecular phylogeny; these parasites are now considered to be separate species in their own right [9,10] (Table 1). A fourth 'large' (as yet unnamed) *Babesia *sp. has been described recently in a number of dogs with clinical signs and haematological parameters consistent with babesiosis in North Carolina [11,12]. With regard to small piroplasms, three genetically and clinically distinct species are currently recognised to cause disease in dogs; *Babesia gibsoni*, *Babesia conradae *(reported in dogs in the western United States and described in original reports as "*B. gibsoni*") [13,14], and a *Babesia microti*-like piroplasm (named *Theileria annae*) [15,16].

**Table 1:** Piroplasm species of domestic dogs.

Size	Species	Synonyms	Vector in dog	Geographic Distribution	Comments
Large	*Babesia vogeli*	*Babesia canis vogeli*	*Rhipicephalus sanguineus*	Wide range:Tropical, subtropical and Mediterranean regions	
	*Babesia canis*	*Babesia canis canis*	*Dermacentor *spp.	Europe	
	*Babesia rossi*	*Babesia canis rossi*	*Haemaphysalis elliptica *(formerly *H. leachi*)	Sub-Saharan Africa, South Africa	
	*Babesia *sp.	Un-named large *Babesia *sp., North Carolina isolate	Unknown	North Carolina, USA	
Small	*Babesia gibsoni*	*Babesia gibsoni *Asia strain	*Haemaphysalis longicornis*	Asia including Japan, sporadic occurrence worldwide	Outside Asia this infection is often associated with Pit Bull Terriers and other fighting dogs
	*Theileria annae*	*Babesia microti*-like Spanish isolate/piroplasm/agent	*Ixodes hexagonus *(putative)	Spain, Portugal	
	*Theileria *sp.	Un-named *Theileria *sp., South African *Theileria *sp.	Unknown	South Africa	Molecular detection only
	*Theileria annulata*		Unknown	Africa, Europe, Asia	Molecular detection only
	*Theileria equi*	*Babesia equi*	Unknown	Africa, Europe, Asia	Molecular detection only

In addition, three *Theileria *species have been isolated in a small number of dogs' blood in Europe (*Theileria (Babesia) equi *and *Theileria annulata*) [17,18] and from 82 dogs in South Africa (unnamed *Theileria *sp. related to an isolate obtained from antelope) [19] (Table 1). Until more information is available the competence of the dog as a host for these piroplasms is uncertain; the clinical correlation for these organisms is unknown, and neither intraerythrocytic nor extraerythrocytic stages have yet been visualised.

As with many taxonomic debates, the classification of canine piroplasms has not been without disagreement among parasitologists, especially concerning nomenclature of the smaller parasites. Furthermore, data from molecular analysis of the mammalian host stages have far outstripped our understanding of the life-cycle features of these organisms, notably identification of their vectors and other important biological data. Under light microscopy the intraerythrocytic stage of *Babesia *spp. is indistinguishable from *Theileria *spp. (In cats *Cytauxzoon *is also similar in appearance, but to date there has been no report of this genus affecting canines.) Historically these two genera, *Babesia *and *Theileria*, are separated on the basis of certain life-cycle stages and transovarial passage within the tick vector [20], yet to date the only piroplasm of companion animals known to have an extra-erythrocytic (schizogony) stage to its life cycle is *Cytauxzoon felis *in the cat.

The controversy focuses on whether all small canine piroplasm species should be classified as *Babesia *spp. or whether *Theileria *spp. infect dogs as part of their natural life cycle. To inform the debate, molecular studies of canine piroplasms have predominantly utilised the small subunit ribosomal 18S gene locus to infer phylogenetic relationships, favoured for its conserved nature and predictable rate of mutation, but other rRNA loci, *cytochrome b*, and genes encoding the heat-shock proteins have been also utilised for this purpose [6,18,21].

As noted above, pre-erythrocytic parasite (schizont) stages in lymphocytes or macrophages, a defining feature of *Theileria*, have not been observed in dogs. However, the formation of distinct tetrad forms (the "Maltese cross"), considered as a feature of both *Theileria *spp. and *Babesia microti*, has been described in *Babesia conradae *[14,22]. *Babesia conradae *appears to be most closely related to a group of piroplasms found in free-ranging ruminants (deer and sheep) and isolated from humans in the western United States – the 'western clade' [22]. This group is phylogenetically distinct from *Babesia microti*, a rodent piroplasm with a wide distribution throughout the Holarctic ecozone (see review [23]) in which schizogony in lymphocytes has been reported [24]. The piroplasm species reported in dogs from northern Spain forms into a clade with *Babesia microti *and was given the name *Theileria annae *[15], but is often referred to as the "*Babesia microti*-like" piroplasm or agent, or "Spanish isolate". Indisputably *Theileria *species (*Theileria annulata *and *Theileria equi*) have been detected in canine blood [17,18] and the most recently reported canine piroplasm, in South Africa [19], is also phylogenetically aligned with recognised *Theileria *spp., yet as stated previously their significance is unclear at the present time.

## Geographical distribution

The distribution maps for canine vector-borne diseases are continually changing as new information about parasite infections and ranges comes to light. In general it may be useful to consider two levels of regional prevalence and incidence for specific diseases; those regions where the specific parasite is well established (i.e., endemic) and clinically recognised; and those regions where sporadic autochthonous infections or cases associated with travelling dogs have been reported.

### Established endemic ranges

The established ranges for canine piroplasms are listed in Table 1; comprehensive and up-to-date canine vector-borne disease maps of Europe, and East and Southeast Asia are available [25]. As would be expected, the geographical distribution of these organisms is delineated largely by the ecological ranges of their vector ticks. Interestingly this generalisation seems not to apply to *Babesia gibsoni*, whose rapid recent global dispersion is now proposed to have been achieved predominantly by direct dog-to-dog transmission under unique ownership and management practices, without involvement of a vector (see later discussion) [4,5].

*Babesia vogeli *is the most widespread canine piroplasm due to the cosmopolitan nature of its host, the brown dog tick *Rhipicephalus sanguineus *(Table 1). *Babesia vogeli *has a truly worldwide distribution throughout tropical and subtropical regions and extending into cooler latitudes where it potentially occurs alongside (and may be confused with) the other large *Babesia *spp. *Babesia canis *(*sensu strictu*) is transmitted by *Dermacentor *spp. and has been increasingly recognised throughout central Europe when it was once thought to be confined to France. *Babesia rossi *is the other African *Babesia *(with *B. vogeli *and *B. gibsoni*); originally recognised only in South Africa but has been reported recently in other regions of the African continent including Nigeria [26] and Sudan [27] where its vector ticks (*Haemaphysalis *spp.) are enzootic (Table 1).

The small canine piroplasm with the greatest geographical distribution is undoubtedly *B. gibsoni*. The type-species is referred to as the "Asia strain", reflecting its original molecular identification from a number of different southern, eastern and southeastern Asian countries. However, in the last 10 years *B. gibsoni *infections have been reported in many countries outside Asia, in American Pit Bull Terrier-type dogs predominantly, and there is now convincing evidence that these cases have arisen due to biting and fighting between infected and non-infected dogs [4,28]. Gene sequence conservation has been noted in the ITS and 18S gene of *B. gibsoni *isolates originating in many disparate regions of the world [6,22,29]. This observation has led to the intriguing hypothesis that the lack of genetic diversity in *B. gibsoni *is a result of the absence of sexual reproduction (sporogony) in the tick; in other words a clonal expansion of a single strain is occurring within a susceptible host population. Therefore the dogs themselves are the reservoir for *B. gibsoni*, specific fighting dogs in particular, and not an endemic species of tick. Due to the worldwide popularity of this and similar breeds, it is speculated that *Babesia gibsoni *will be reported eventually from all countries where (usually illegal) dog fighting is practiced.

The three most recently characterised small piroplasm species of dogs occur, as previously mentioned, in the western United States, specifically California (*Babesia conradae*), southern Africa (*Theileria *sp.) and the Iberian peninsular (*Theileria annae*), where the latter is thought to be transmitted by *Ixodes hexagonus*. The vectors for *Babesia conradae *and the South African *Theileria *sp. are currently unknown and apart from a single case report of *Theileria annae *in the USA [28], these piroplasms have not been reported outside these fairly localised geographical boundaries.

### Autochthonous infections and other single infection reports

Autochthonous infection refers to discovery of an infection that has originated in the place where it is found, usually in the context of a new, or unexpected finding in a location where the infection is generally not considered to occur. In the case of babesiosis, autochthonous cases are most likely to arise when dogs have come into contact with infected vectors which may themselves have been introduced by hosts returning from vector-endemic areas, or by extension of the vector range due to ecological changes. There have been a number of reports of autochthonous babesiosis and piroplasmosis in northern Europe (where vector-borne diseases have been considered unusual or exotic) and such cases have raised concern among the local veterinary and pet-owning fraternities. The importance of collecting travel history as an integral step during a veterinary consultation has been emphasised, together with concern about the risks of disease spread associated with deregulated pet travel, particularly in mainland Europe and the UK. The history of being a hunting dog or extensive rural exposure is a risk factor for canine babesiosis in Europe [30].

## Diagnosis of babesiosis

Microscopy remains the simplest and most accessible diagnostic test for most veterinarians and during acute infections microscopy is reasonably sensitive for detecting intraerythrocytic parasites in Giemsa or Wright's stained blood smears. Differentiation between large and small piroplasms is also relatively simple. Moreover, microscopy is still the only viable option available to veterinarians in many parts of the developing world where babesiosis is endemic. With large *Babesia *species at least, sampling from capillary beds (ear tip, toe nail) or examination of cells from beneath the buffy coat of a haematocrit tube may improve the probability of finding parasites [31,32].

The diagnosis of piroplasmosis in chronically infected and carrier dogs however remains a significant challenge due to very low, often intermittent parasitaemias. Failure to detect *Babesia/Theileria *parasites in animals with hemolytic anaemia or thrombocytopenia has led to an incorrect diagnosis in documented cases, often when the clinical suspicion of babesiosis was also low. Given the possibility of direct horizontal transmission of *Babesia gibsoni*, veterinary clinicians should always ascertain whether the patient has been bitten by any other dog in the preceding 4–8 weeks, irrespective of its breed [4,28].

### Molecular diagnosis – conventional PCR and other methods

Although PCR has greatly increased the sensitivity and specificity of parasite detection and is well suited to epidemiological and phylogenetic studies, access to molecular techniques for routine clinical diagnosis of babesiosis is still restricted to relatively few laboratories worldwide. Ribosomal RNA genes 18S, 5.8S, 28S and the internal transcribed spacer (ITS) sequences have been used for conventional PCR, but some researchers have chosen other loci such as the p18/BgTRAP [33]. Since parasite morphology is a poor guide to speciation, modifications of the PCR have been utilised to rapidly differentiate between piroplasm species; PCR-RFLP and nested PCR have been reported to differentiate *B. vogeli *and *B. gibsoni *in Australia [34], and between the large babesial species [9] and *B. gibsoni *in endemic regions [35]. In a clinicopathological study of large *Babesia *spp. infections in dogs in Italy using PCR-RFLP, *Babesia canis *was detected in 34/164 and *Babesia vogeli *in 11/164 dogs. Although a distinct geographical difference in the incidence of these infections is noted [30], many of the dogs with *B. canis *infection had recently returned from hunting trips in Eastern Europe. Further refinement in primer design was reported recently to clearly separate amplicons of 342 bp, 546 bp, and 746 bp target fragments of *B. canis rossi, B. canis vogeli, B. canis canis*, respectively [36]. Loop-mediated isothermal amplification (LAMP) was found to have advantages of speed and specificity for detecting *B. gibsoni *infections in Japan [37] and reverse-line blot (RLB) hybridisation was applied in epidemiological studies of arthropod-borne haemopathogens of dogs and cats in Trinidad [38] and dogs in Africa [19]. Real-time PCR enables quantification of pathogen levels within blood and tissue samples; the amount of final PCR product can be used to deduce the starting number of target molecules and infer parasite levels within a host. Quantitative PCR (qPCR) was used in an experimental infection of 3 dogs with *B. gibsoni *that concluded that the methodology might be adapted to the determination of vaccine or chemotherapeutic efficacy, or the elucidation of immunological responses [39]. Furthermore, a number of these PCR methods have been applied to filter-paper technologies such as FTA cards (Whatman Bioscience) and IsoCode Stix (Schleicher and Scheull) for ease of transport of samples to distant laboratories and for epidemiological and other diagnostic studies [34,40]. Long-term storage and archival qualities of this methodology have not been investigated [40].

Whereas the detection limit of light microscopy is approximately 0.001% parasitaemia, PCR is capable of detecting parasite loads in the region of 50 organisms/ml [35] and 9 parasites/μl [39]. Yet despite its extraordinary sensitivity, PCR will clearly not detect target DNA when there are no organisms within the sample. "False negative" results may occur in chronic babesiosis and it is very important to recognise this limitation when screening potential carriers and other asymptomatic dogs such as blood donors. The ability of PCR to detect infected dogs in such situations, with and without treatment, has been investigated [41,42]. In one study [42] clinical parameters, haematology, serologic titer (by IFAT) and the presence of *Babesia *DNA was monitored on a daily basis after experimental infection. All dogs (n = 3) made a full clinical recovery, as judged by normal clinical signs, absence of splenic enlargement, a normal haemogram and absence of piroplasms on microscopic examination by 30–50 days after peak parasitaemia. During this period of clinical normality, babesial DNA was inconsistently detected. This suggests a very low, fluctuating parasitaemia in these dogs, possibly analogous to chronic, asymptomatic natural infection.

The ability of PCR to detect parasite DNA in chronically infected animals can be improved by testing on more than one occasion, but the use of serology as an alternative, complementary diagnostic test is advisable in these situations [42,43].

### Serological testing – the IFAT and ELISA

Immunofluorescent antibody testing (IFAT) has been the most widely supported serological diagnostic test for canine babesiosis for the last 30 years [44,45]. However, poor specificity due to cross-reactions between *Babesia *spp. and with other apicomplexan parasites, operator subjectivity and its inadequacy for large-scale screening have all been limiting factors [46]. Recent research by several groups in Japan directed towards finding specific immunodominant *Babesia gibsoni *antigens for use in recombinant protein enzyme-linked immunosorbent assays (ELISA) – and potential vaccine candidates in the future – has identified numerous promising compounds [46-52]. Thrombospondin-related adhesive proteins (TRAPs) comprise a group of highly conserved functional proteins identified in apicomplexan parasites, mooted to be associated with merozoite motility and invasion, and capable of inducing a host antibody response [49]. An ELISA using recombinant BgTRAP was reported to be more sensitive than other ELISAs using recombinant antigens rBgP50, rBgSA1, and rBgP32 [43], and Konishi et al. (2008) utilised a BgTRAP ELISA to test 1,206 randomly selected non-fighting breed dogs in Japan to test for exposure to *B. gibsoni *[53]. These authors reported higher infection rates in western Japan and concluded that having excluded fighting dogs, a history of tick (*Haemaphysalis longicornis*) exposure was a significant risk factor for anti-babesial antibody detection, thus demonstrating two distinct epidemiological patterns of *B. gibsoni *infection in that country.

A second major limitation of serological tests is their inability to differentiate acute from chronic infections, and interpretation of a positive titre is somewhat problematic for clinicians working in regions that are endemic for babesiosis. Nevertheless, for *B. gibsoni *in the USA and Australia, where cases are sporadic, the IFAT is a useful tool for detection of infected dogs, especially if combined with PCR [31,38].

## Pathophysiology

The severity of babesiosis in dogs and cats ranges from subclinical infection, the development of mild anaemia to widespread organ failure and death. The critical determinant of this variable pathogenesis is the piroplasm species, yet other factors such as the age and immune status of the host and concurrent infections or illness also play a role. All species (Table 1) may cause pyrexia, anorexia, splenomegaly, anaemia and thrombocytopenia. Direct parasite-induced red-cell damage, increased osmotic fragility of infected cells, oxidative and secondary immune-mediated injury of the erythrocyte membrane result in a combination of intravascular and extravascular haemolysis.

The clinical features of babesiosis have been reviewed elsewhere [1-3]. In broad terms it is generally agreed that the least pathogenic of the well-recognised canine piroplasm species is *Babesia vogeli*, at least in adult dogs, and the most virulent is *Babesia rossi *in Africa [1,30,31]. With *Babesia rossi *infections a large proportion of dogs develop complications, some of which (hepatopathy, immune-mediated haemolysis) typically extend hospital stay but do not affect mortality if treated appropriately, while others (haemoconcentration, neurological signs, acute renal failure and pulmonary oedema) require early, aggressive and intensive therapy and carry a poor prognosis [32]. In contrast, *Babesia vogeli *is often subclinical (except in puppies less than 3–4 months old, in which infection may be fatal); it is occasionally observed in blood films of dogs with other primary disease or receiving medical treatment (e.g., immunosuppression or chemotherapy) or surgery (notably splenectomy), and in these potentially immunocompromised individuals the appearance of the parasite may be inconsequential to the outcome (and may not even warrant treatment).

The pathogenicity of *Babesia canis, Babesia gibsoni, Theileria annae *and *Babesia conradae *is moderate to severe in dogs, but it should be stressed again that a wide range of clinical signs of varying severity can be observed in individuals. *Babesia conradae *is considered to be more pathogenic than *B. gibsoni*, resulting in higher parasitaemias and more severe anaemia [14]. In Spain, *Theileria annae *infection is associated with severe haemolysis and azotaemia [16]. The unnamed *Babesia *sp. from North Carolina has been associated with non-specific illness (lethargy and anorexia), pigmenturia and mild fever, predominantly in splenectomised dogs [54]. Laboratory findings have included mild anaemia and severe thrombocytopenia, similar to *Babesia vogeli *infections [54].

The potential for renal involvement in dogs with babesiosis has received attention in recent years, with hypoxaemia, haemoglobinuric nephropathy and glomerulonephritis all considered possible mechanisms and supported by histological studies [55]. A clinicopathological study of *Theileria annae *infection in Northwest Spain reported 36% dogs (n = 58) were azotaemic at the time of diagnosis and that these dogs had a 10-fold higher risk of death from piroplasmosis than those that were not azotaemic [56]. Unfortunately neither urine-specific gravity nor urine osmolality was reported in these dogs, but elevated urine protein: creatinine ratios, hypoalbuminaemia and hypercholesterolaemia in affected dogs led the authors to suggest that glomerular injury was occurring and that renal failure was most likely a feature of *Theileria annae *infection [56]. In contrast, study of azotaemia associated with *B. rossi *infection concluded that urea and creatinine were unreliable indicators of renal damage or acute renal failure in acute babesiosis [57,58]. Mortality of *B. rossi *was significantly associated with high cortisol and high ACTH concentrations and with low T4 and fT4 concentrations in a recent study investigating endocrine markers of disease [59].

The clinical consequences of chronic babesial infection are unclear and while most dogs appear to tolerate this state of premunity with few ill effects, theoretically they remain at risk of developing immune-mediated complications and recrudescence of clinical disease (and parasitaemia) if immunocompromised at a later time. Chronic infection may be inconsequential in some dogs and may be even beneficial for hosts living in endemic regions by protecting them from further disease [60].

## Recent advances in the treatment and prevention of babesiosis

Despite a plethora of anecdotal reports and uncontrolled experimental data, there is a paucity of scientifically robust evidence regarding the efficacy of drugs that have been used to treat canine babesiosis over the last 100 years. Early treatment studies were hampered by the need to rely on clinical signs and blood films to determine parasite clearance. More recently the limitations have been the cost of establishing controlled infections and the relatively small numbers of experimental dogs permitted by animal ethics committees. In order to determinate the efficacy of an anti-piroplasm drug with reasonable confidence, necropsy-obtained tissues from all organs need to be tested by validated PCR at the end of the experiment; clearance of the parasite DNA from peripheral blood alone is insufficient evidence of cure.

Imidocarb dipropionate and diminazine aceturate are widely used anti-piroplasm drugs, but other compounds that have been used for many years and have enjoyed varying degrees of success in managing the clinical signs of piroplasmosis include quinuronium sulphate, trypan blue, pentamidine, phenamidine and parvaquone. National registration authorities have restricted access to some of these drugs in certain countries, and some, notably the diamidine derivative diminazine, are associated with a high rate of toxic side effects. At best these drugs result in amelioration of clinical signs; rarely do they achieve true sterilisation of the infection.

The successful treatment of the small piroplasm infections, notably *B. gibsoni*, has been especially challenging. Clindamycin at 25 mg/kg q12 h PO induced morphological changes and reduced the parasitaemia in an experimental infection compared with untreated controls, but did not eliminate the parasite, and piroplasms were still observed in low numbers 108 days after infection [61]. Recently, the apparent cure of *B. gibsoni *was reported in 3 out of 4 experimental dogs that had not responded to repeated diminazine treatment, with a combination of clindamycin, metronidazole (15 mg/kg q12 h PO) and doxycycline (5 mg/kg q12 h PO) [62]. Successful treatment in these dogs was determined by a combination of normal clinical signs and the absence of *B. gibsoni *DNA in peripheral blood. More recently, a combination of azithromycin (10 mg/kg q24 h PO) and atovaquone (13.3 mg/kg q8 h PO) for 10 days has been used for treating *B. gibsoni *and appears to combine reasonable clinical efficacy with great safety [41]; the expense of atovaquone limits widespread acceptance of this therapy where it is most needed, in Asia. Cheaper formulations of atovaquone with proguanil cause an unacceptably high incidence of gastrointestinal side effects in dogs. Unfortunately this drug combination also does not result in a cure in some dogs, and rapid development of resistance to atovaquone caused by mutation of the *cytochrome b *gene has been reported [42,63].

*In vitro *studies of numerous rainforest plant extracts for their antibabesial properties have been published recently [64-66], but to date no clinical trials have been reported with these compounds. A *Haemaphysalis longicornis *tick-derived peptide reduced parasitaemias of *B. microti *in BALB/c mice and directly kills *B. gibsoni *parasites in ticks [67].

Prevention of babesiosis, as with any tick-transmitted disease, is best achieved by removing the possibility of exposure to the vector. This is rarely achievable in endemic areas despite attentive ectoparasite control.

Several drugs have been investigated for their prophylactic potential against babesiosis, yet none has been consistently reliable in this regard. Experimental studies suggested that a single dose of imidocarb dipropionate (6 mg/kg) protects dogs from *B. canis *challenge for up to 8 weeks [68] and that doxycycline at 5 mg/kg/day ameliorates the severity of disease when challenged with virulent *B. canis *[69].

*In vitro *culture-derived soluble parasite antigens (SPA) have been used to protect dogs in European against *B. canis *(*sensu strictu*) challenge in a commercially available vaccine since the 1980s, and it was reported in one study that the incidence of babesiosis decreased from 16% to near zero in populations of vaccinated dogs living in endemic regions over the three-year period of study (reviewed in [70]). However, variable efficacy of the SPA vaccine during homologous challenge has been attributed to strain variation [71] and it was clear from early studies that *B. canis*-derived SPA did not protect dogs from heterologous challenge with *B. rossi *[72], which led to the development of a vaccine containing a mixture of SPA from both European *B. canis *and South African *B. rossi *that offered greater protection against heterologous challenge [73]. Current research is also conducted in Japan in search of suitable immunodominant and protective antigens for use in vaccines against *Babesia gibsoni *[74].

## Conclusion

Canine babesiosis/piroplasmosis remains as a significant disease that despite considerable advances during the last decade in our knowledge and understanding of the pathogens themselves, the intricacies of transmission, and their pathophysiological mechanisms, still poses significant diagnostic and therapeutics challenges for veterinary practitioners around the world. New species of piroplasm will almost certainly be described, and the geographical range of established piroplasms will expand due to international movements of dogs and expansion of vector tick habitats. The challenges for the researchers are to provide practitioners with readily accessible and accurate diagnostic tools, safer and more efficacious anti-babesial drugs, and the "Holy Grail" of this applied research – effective immunologicals for the prevention or, at least, amelioration of the clinical signs of canine babesiosis.

## Competing interests

The author declares that they have no competing interests.
